# Lung and Intestine: A Specific Link in an Ulcerative Colitis Rat Model

**DOI:** 10.1155/2013/124530

**Published:** 2013-03-28

**Authors:** Yuan Liu, Xin-Yue Wang, Xue Yang, Shan Jing, Li Zhu, Si-Hua Gao

**Affiliations:** ^1^Department of Internal Medicine of TCM, Dongzhimen Hospital, Beijing University of Chinese Medicine, 5 Hai Yun Cang, Dongcheng District, Beijing 100700, China; ^2^Department of Internal Medicine of TCM, The Third Hospital affiliated with Henan University of Traditional Chinese Medicine, 63 Dong Ming, Jinshui District, Zhengzhou, Henan 450008, China; ^3^Department of Internal Medicine of TCM, Nantong Hospital, 41 Jianshe Road, Chongchuan District, Nantong, Jiangsu 226000, China; ^4^Department of Basic Theory of TCM, School of Preclinical Medicine, Beijing University of Chinese Medicine, 11 Bei San Huan Dong Road, Chaoyang District, Beijing 100029, China

## Abstract

*Background*. To investigate the link and mechanisms between intestine and lung in the ulcerative colitis (UC) rat model. *Materials and Methods*. We used the UC rat model by immunological sensitization combined with local 2, 4, 6-trinitrobenzene sulfonic acid (TNBS) in 50% ethanol enema, observed dynamically animal general state and body weight, examined the histological and functional changes in the colon, lung, liver, and kidney tissues, and detected microvascular endothelium response towards inflammation characterized with the expression of iNOS, TXB_2_, P-selectin, ICAM-1, and vascular endothelial growth factor A (VEGF-A) in the colon and lung tissue. *Results*. Pulmonary function results suggested ventilator disorder, and pathological findings showed interstitial pneumonia. There were no significant changes in the liver and kidney function and histopathology. The colon and lung tissue iNOS, TXB_2_, P-selectin, ICAM-1, and VEGF-A expression of the model rats was significantly higher than the normal rats at both time points. *Conclusions*. Our study is the first to demonstrate the close association between the large intestine and lung in the immune-TNBS-ethanol-induced UC rat model. Different organs and tissues with the same embryonic origin may share the same pathological specificities in a disease. The present study provided a new way of thinking for pathological changes in clinical complex diseases manifested with multiorgan damage.

## 1. Introduction

Inflammatory bowel disease (IBD) is a term that describes a group of inflammatory diseases of the gastrointestinal tract. Ulcerative colitis (UC) is one of the most common forms of IBD [1]. UC is characterized by repeated cycles of colonic mucosal ulceration and regeneration and associated with excessive daily bowel movements, severe abdominal pain, diarrhea, weight loss, malnutrition, and intestinal bleeding [2]. Although the precise etiology of UC remains unknown, the impairment of the microvascular endothelial barrier has been recently shown to play important pathophysiologic role [3–5].

UC also may have many extraintestinal involvements during the process of disease. The pulmonary abnormality may represent one of the extraintestinal disorders. Turner-Warwick [6] described a possible association of UC and respiratory disease in 1968. In 1976, respiratory involvement was included in the list of established complications of IBD by Kraft et al. [7]. Since then, a number of studies have described various patterns of pulmonary involvement [8–12] in IBD, and it is believed that 50%–60% of IBD patients have subclinical pulmonary abnormalities [13–15]. The most prevalent abnormality is a reduction in a gas transfer (transfer coefficient for carbon monoxide DLCO) of about 50% [14] and an elevation of the residual volume : total lung capacity (RV : TLC) ratio [15]. An increasing disease activity is associated with abnormal pulmonary function, suggesting a direct pathogenic link to IBD [15, 16]. Although the exact mechanism is unknown, reports have shown that there is a shift of the inflammatory process from the bowel to the lung, perhaps related to the common ancestry of the bowel and the bronchial tree [14, 15, 17]. Additionally, the gut-associated lymphoid tissue shares adhesion molecules that are involved in the homing of leukocytes to both gut and bronchiolar lymphoid tissues [14, 15].

However, there is little data to show the link between large intestine and lung in animal models. Animal models of UC, therefore, offer an invaluable tool to study the pathogenesis and possible etiology of this disease. The quality of animal models has a direct impact on the findings. There are various experimental animal models of UC currently available including those chemically induced (2, 4, 6-trinitrobenzene sulfonic acid (TNBS), dextran sodium sulfate, and acetic acid), those that have been genetically manipulated (interleukin [IL]-10 knockouts), and other models that are used for various research purposes [18–23]. Although no animal model completely resembles the human condition, the UC rat model by immunological sensitization combined with local TNBS-50% ethanol irritation appears to present with many symptoms similar to those seen in humans. This model is characterized by reproducible intestinal mucosal barrier damage and immune imbalance which are the most important risk factors for UC patients. Antigen preimmunization causes rat systemic sensitization and immune abnormalities [19]. Ethanol is required to break the mucosal barrier, whereas TNBS is believed to haptenize colonic autologous or microbiota proteins rendering them immunogenic to the host immune system. The colonic TNBS enema results in an acute local inflammatory response (colitis because it is located in the colon) with imbalance in Th1 cytokines (IFN-*γ*, TNF-*α*, and IL-12) and Th2 cytokines (IL-4, IL-5, and IL-13) [24–26]. It quickly develops to a long-lasting damage characterized by inflammatory cell infiltration and ulcers. The rat is a good choice to study UC because it has been shown to possess a similar colonic morphology; the inflammatory parameters observed with healing are similar to those found in humans; it is easy to manage with low mortality [27]. Here, we present further evidence supporting the immune-TNBS-ethanol-induced UC rat model as an appropriate tool to study the UC pathology and identify the link between large intestine and lung.

Although the links between large intestine and lung have been clearly identified in the clinical literature, there have been few basic research studies that have investigated the mechanisms of the inflammatory cross-talk involved. It has now been clearly established that the microvascular changes associated with angiogenesis are key contributors to the tissue injury and remodeling process that inevitably accompanies chronic inflammation. Vascular endothelial growth factor A (VEGF-A) is a fundamental mediator of IBD by promoting intestinal angiogenesis and inflammation, and targeting VEGF-A is a promising new therapeutic strategy to dampen inflammation [5]. In this study, we investigated the role of the microvasculature in the colon and lung tissues of UC rats to further our understanding of the theory of the large intestine and the lung that have a close internal connection and provide further evidence for new treatment strategies that target the microvascular endothelium. 

## 2. Materials and Methods

All chemicals were purchased from Sigma laboratories (St. Louis, MO, USA) unless stated otherwise.

### 2.1. Animals

Male Wistar rats weighing 200 ± 10 g were purchased from the Academy of Military Medical Laboratory Animal Centre (Beijing, China). Ten male New Zealand rabbits weighing about 3 kg were purchased from Haidian Thriving Animal Farm (Beijing, China). The animals were housed at SPF Animal Laboratory of Dongzhimen Hospital (Beijing, China) with free access to food and water and were kept in a regulated environment (22 ± 2°C) under a 12-hour light/dark cycle (light on at 8:00 am). All animal procedures were performed strictly within national regulations and guidelines and approved by the Animal Experimentation Committee at Beijing University of Chinese Medicine. The rats were randomly divided into four groups as follows: normal_24 h_ (*n* = 10); model_24 h_ (*n* = 15)—immune-TNBS-ethanol-induced UC rats, for 24 hours; normal_4 w_ (*n* = 10); model_24 w_ (*n* = 15)—immune-TNBS-ethanol-induced UC rats, for 4 weeks.

### 2.2. Induction of Ulcerative Colitis

Antigen preparation: ten rabbits were sacrificed by air embolism and immediately removed the colon. The colon was rinsed with sterile saline and scraped the mucosa. The mucosa was mixed with equal saline and homogenized 30 times with a tight Dounce homogenizer (Sigma, USA). Samples were further disrupted by intermittent sonication (six 30 s pulse with a 1 min cooling period in between) and then centrifuged at 3,000 rpm (Eppendorf, Germany) for 30 min at 4°C. The supernatant was then aliquoted and stored at −20°C. The protein concentration was measured using bicinchoninic acid (BCA) assay (CoWin Bioscience, China). 

**Figure 1:**
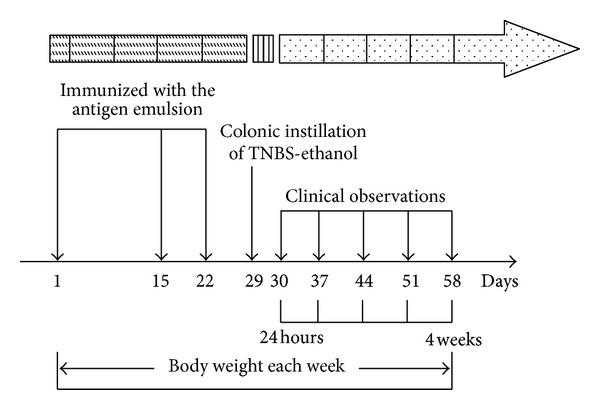
Schematic representation of study design. On the day 1, day 15, and day 22, the antigen emulsion was immunized in the paws and groin. On day 29, TNBS-ethanol was instilled into the colon. Clinical observation was performed after 24 hours of TNBS-ethanol enema for 4 consecutive weeks. Rats were weighed every weekend. On the end of 24-hour or 4-week administration, rats were sacrificed, respectively.

Antigen preimmunization and TNBS enema: Rabbit intestinal mucosal antigen solution was mixed with equal volume of Freund's complete adjuvant and made of the antigen emulsion. Wistar rats were immunized with the antigen emulsion in the paws, groin, and other parts at day 1, day 15, and day 22, respectively. Each immune volume contained antigen protein 8 mg per rat. On day 29, rats were anesthetized with 10% chloral hydrate (0.4 mL·kg^−1^, i.p.), and then a medical-grade polyurethane cannula for enteral feeding (external diameter 2 mm) was inserted into the anus, and the tip was advanced to 8 cm proximal to the anal verge. TNBS dissolved in 50% ethanol was instilled rapidly into the colon through the cannula at 100 mg·kg^−1^ and the animals were maintained in a head-down position for a few minutes to prevent leakage of the intracolonic instillation. Rats were killed at day 1 (24 hours after TNBS-ethanol enema) and day 28 (4 weeks after TNBS-ethanol enema) (Figure 1).

### 2.3. Histological Analysis

Paraffin-embedded sections were stained with hematoxylin-eosin (HE) and masson-trichrome (MT) to evaluate general morphology and collagen formation. The tissue of 8 cm colon (between 10 cm distal to the anus and 2 cm proximal to the anus), lung, liver, and kidney were, respectively, removed, and washed in saline. HE and MT staining were done as previously described [28, 29]. To demonstrate the expression and localization of iNOS, ICAM-1, and VEGF-A, immunohistochemistry was used as previously described [28]. The colon and lung sections were incubated with iNOS (anti-rabbit, 1 : 50, Abcam), ICAM-1 (anti-mouse, 1 : 100, Abcam), or VEGF-A (anti-rabbit, 1 : 250, Abnova) and detected, respectively, with the secondary antibodies (CoWin Bioscience, China).

### 2.4. Liver and Renal Function Analysis

At the end of the experiment, rats were deprived of food overnight and anesthetized with 10% chloral hydrate. The abdomen was sprayed with 70% ethanol and cut open. The viscera were displaced to one side, and the tissues covering the dorsal aorta were reflected with forceps. The abdominal aorta was punctured with a 2 mL syringe, and the blood was collected into a 1.5 mL tube. 1 mL of blood was collected from each rat. The blood was allowed to clot for about 3 hours at room temperature and then centrifuged at 3,000 rpm for 15 min at 4°C. The serum was removed, aliquoted, and stored at −80°C. The serum concentration of alanine aminotransferase (ALT), aspartate aminotransferase (AST), blood urea nitrogen (BUN) and creatinine (Cr) was detected by clinical laboratory of Dongzhimen Hospital affiliated with Beijing University of Chinese Medicine (Beijing, China).

### 2.5. Pulmonary Function Test Analysis

Pulmonary function tests were conducted on five rats from each group at Respiratory Foundation Lab of Chaoyang Hospital (Beijing, China). Animals were anesthetized with 12% urethane (0.2 mL·kg^−1^, i.p.) to surgical depth in a whole body box, and a tracheal cannula (3-way connecting tube, 0.24 cm i.d.) was inserted. The parameters for the pulmonary function tests included the resistance of airways at inspiration (Ri, cm·H_2_O/mL·s^−1^), resistance of airways at expiration (Re, cm·H_2_O/mL·s^−1^), lung compliance (CL, %), maximal voluntary ventilation (MVV, mL/min), forced vital capacity (FVC, mL), forced expiratory volume in 0.2 second (FEV0.2, mL), ration of FEV0.2 and FVC in % (FEV0.2/FVC, %), peak expiratory flow (PEF, mL/s), forced midexpiratory flow rate (FEF25%–75%, %). Following the pulmonary function tests, the animals were kept warm, allowed to recover from the anesthetic, and were extubated. They were housed in a laminar hood in standard rat cages with sterile paper pellets as bedding and were allowed free access to food and water.

### 2.6. Western Blotting Analysis

To determine the levels of ICAM-1 and VEGF-A in the colon and lung tissue, western blotting was used as previously described [28, 30]. The membranes were probed with primary antibody to ICAM-1 (anti-mouse, 1 : 1000, Abcam) or VEGF-A (anti-rabbit, 1 : 500, Abnova) and detected with secondary antibodies (1 : 3000, Santa Cruz, USA). *β*-actin was used as a gelloading control. Signals were detected using enhanced chemiluminescence (Perkin-Elmer Life Science), and band intensities were quantified using Image J software (NIH, Bethesda, MD, USA).

### 2.7. Radioimmunoassay Analysis

At the end of the experiment, rats were anesthetized with 10% chloral hydrate and immediately removed the colon and the lung and then rinsed with sterile saline. 100 mg tissue was mixed with 9 volumes of saline and homogenized. Samples were further disrupted by intermittent sonication and then centrifuged at 3,000 rpm for 30 min at 4°C. The supernatant was then aliquoted and stored at −20°C. Samples were detected by Radioimmunoassay Institute of Science and Technology Development Centre affiliated with People's Liberation Army General Hospital (Beijing, China).

### 2.8. Reverse Transcriptase-Polymerase Chain Reaction (RT-PCR) Analysis

The mRNA expression for iNOS was determined by RT-PCR [31]. The sequences of the PCR primers were seen in Table 1. Comparison between different treatment groups was made by the determination of the iNOS/GAPDH ratio of the immunoreactive area by densitometry (MxPro QPCR Software 4.10, Agilent Technologies, USA).

### 2.9. Statistical Analysis

Quantitative data were expressed as mean ± SD. The significance of differences between groups was assessed using one-way ANOVA, post hoc comparisons being made using the nonparametric Dunn multiple comparison test. In all tests, the criterion for statistical significance was *P* < 0.05. Statistical analysis was performed with SPSS software 13.0.

## 3. Results

### 3.1. Animal General State

**Figure 2:**
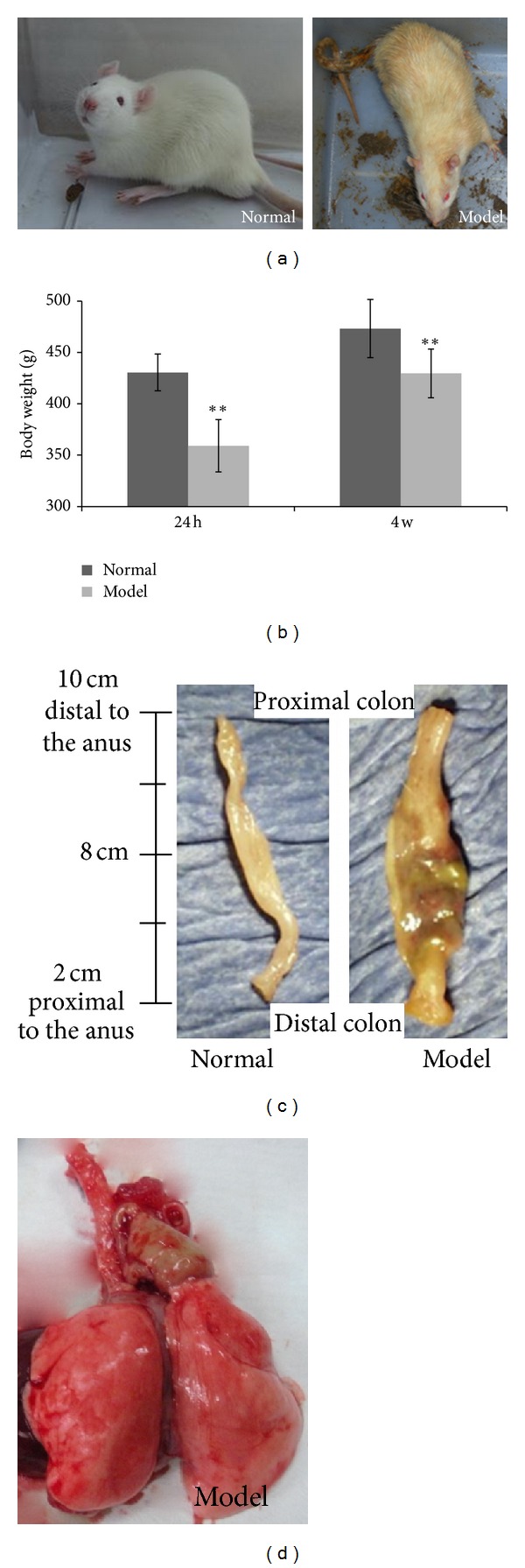
Overall changes in normal and UC rats. (a) Animal general state. Normal rats were active and glossy hair. Feces were hard and granular ellipsoid without mucus and blood. Immune-TNBS-ethanol-induced UC rats were poor, and symptoms such as reduced activity, drowsiness, loss of appetite, slowing response, and emaciation were observed. Hair color was greenish yellow withered. Feces appeared diarrhea mixed with blood, loose or even water. (b) The body weight of the immune-TNBS-ethanol-induced UC rats was significantly lighter than that of the normal rats (**, *P* < 0.01, *n* = 10). (c) Normal operated and immune-TNBS-ethanol-induced UC rat large intestine. Rats were normal or TNBS-ethanol treated for 7 days before sacrifice. Well-advanced inflammation is apparent in the colon of UC rat model. (d) Immune-TNBS-ethanol-induced UC rat lungs. There is an abscess in the left bronchus.

The changes in general state and weight can partially reflect the disease state and recovery of UC rats. Normal rats were agile and active, glossy hair, active foraging, and natural breathing. Feces were hard, granular ellipsoid, dark brown, and without mucus blood. Inflammation became evident in animals received rabbit colonic mucosal antigen emulsion injection. The paws appeared red and swelled. Groin injecting appeared nodules, sporadical suppuration, and ulceration. Some rats had ocular hyperemia. Sickness was also shown as reduced activity, drowsiness, loss of appetite, slowing response, and emaciation. Hair color was greenish yellow withered. Feces gradually became softer, bigger, and more watery. After TNBS-ethanol enema, the feces appeared mucopurulent bloody, loose, or even water on the night of enema. Three days later, it became constant diarrhea mixed with blood, of gradual onset. Respiratory symptoms were also displayed in some animals with appeared breathless and asthmatic wheeze (Figure 2(a)). Pathological examine showed inflamed colon with increased weight and thickness, especially in the distal part (Figure 2(c)). Some rats had abscesses in the bronchus (Figure 2(d)).

Following the TNBS-ethanol enema, rats were weighted every weekend. We found that the body weight of the rats in UC group was significantly less than that of the normal rats (*P* < 0.01). While all animals showed a tendency of weight loss, animals in the TNBS-ethanol enema group weighted significantly less (*P* < 0.01) (Figure 2(b)). 

### 3.2. Specific Correlation in the Large Intestine and Lung but not Other Organs in UC Rats

**Figure 3:**
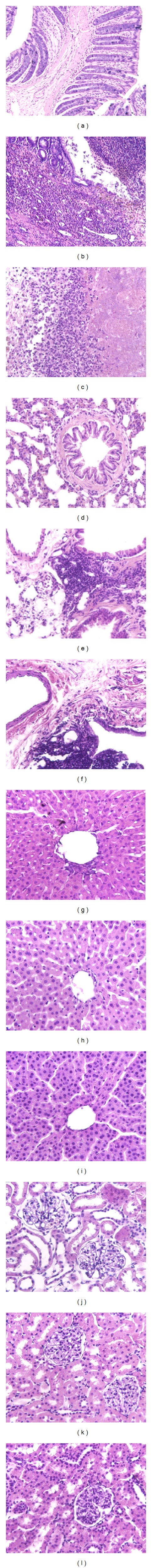
Histological findings in the colon, lung, liver, and kidney of the normal and UC rats (hematoxylin and eosin stain, × 100). (a) The colonic mucosa structure in the normal rats was intact. (b) The colonic mucosa and submucosa defects could be clearly seen with infiltrations of inflammatory cells in the lamina propria of after TNBS-ethanol enema (24 hours). (c) Four weeks later, colonic tissue necrosis was obvious, and some necrotic tissues stripped and formed ulcers. The glands of the lamina propria atrophied or even disappeared. (d) The lung tissue had normal alveolar structure and no inflammatory infiltration and exudate. (e) After TNBS-ethanol enema, the lung tissue can be seen massive lymphocytes, even lymphocytic nodules around the bronchi. (f) Four weeks later, blood vessel wall and bronchial wall were obvious proliferation. (g)–(i) Rat liver structure was basically normal ((g): normal group, (h): model_24 h_ group, and (i): model_4 w_ group). The structure of hepatic cords was clear, liver cells distributed radially around central venous and less fat vacuoles, and the structure of hepatic lobules was intact. (j) The histological appearance of the normal kidney was shown. (k) and (l) Compared with the normal group, glomerular cells and mesangial matrix of the model rats had no significant necrosis and hyperplasia.

Histological examination of tissue sections of the colon, lung, liver, and kidney were performed using HE and MT staining as described previously. As the results of HE staining shown in Figure 3(a), the colonic mucosa structure in the normal group was intact. Mucosa and submucosa defects could be clearly seen with infiltrations of inflammatory neutrophils and lymphocytes in the lamina propria of the model group (Figures 3(b) and 3(c)). After 24 hours of TNBS-ethanol enema, tissue necrosis was obvious and some necrotic tissues stripped and formed ulcers. Ulcer surface was covered with necrotic tissues and newly formed granulation tissue. The glands of the lamina propria atrophied or even disappeared. Blood vessels of submucosa and muscularis dilated. Four weeks later, there was no significant change in the intestinal pathology of the model rats, except the area of ulcer surface expanded. 

The normal histology of the lung is shown in Figure 3(d). The lung tissue had normal alveolar structure and no inflammatory infiltration and exudate. After TNBS-ethanol enema, there were massive lymphocytes, even lymphocytic nodules around the bronchi in the lung of model animals. The lung septum was thickened, while the alveolar septum was narrowed (Figure 3(e)). Four weeks later, there was no further change in the lung pathology of the model rats, besides blood vessel wall and bronchial wall were obvious proliferation (Figure 3(f)). As shown in Figures 3(g)–3(i), rat liver structure of model groups was basically consistent with the normal group. The structure of hepatic cords was clear, liver cells distributed radially around central venous and less fat vacuoles, and the structure of hepatic lobules was intact. The kidney also showed normal histology by 4 weeks in model animals (Figure 3(j)). No obvious necrosis or hyperplasia was seen in the glomerular cells and mesangial matrix of the model rats. No lesions were seen in the tubular, interstitial and blood vessels (Figures 3(k) and 3(l)). 

**Figure 4:**
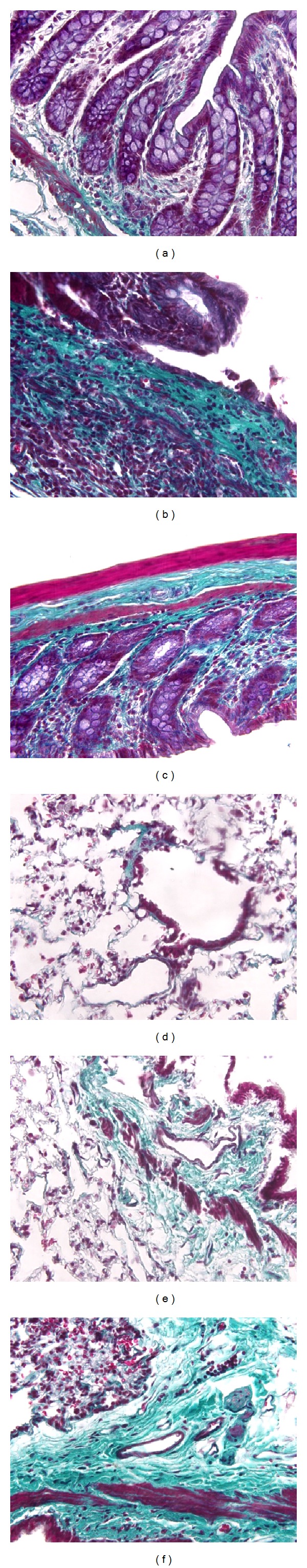
Collagen deposition in the colon and lung of the normal and UC rats (Masson's trichrome stain, × 40). (a) and (d) The intestinal mucosa and bronchia of the normal rats, only a small amount of collagen tissue. (b) and (e) After TNBS-ethanol enema, collagen fibers increased more than normal group, accompanied with red blood cell exudation and bronchial smooth muscle proliferation. (c) and (f) Four weeks later, a large number of collagen fibers proliferated, even replaced the normal mucosa and glands in the colon and lung tissue.

MT staining was used to label collagen fibers and collagen deposition. Differing from HE staining, the MT staining is able to differentiate clearly the important morphological keys for the colon and lung tissue of UC assessment such as haemoglobin, cellulose and muscle fiber (red color), cytoplasm and adipose cells (light red or pink), cell nuclei (blue black), elastic fiber (brown to dark brown), and collagen fiber which stained green in color (Figure 4). The intestinal mucosa and bronchia of the normal rats were normal, and there was no inflammatory cell infiltration, only a small amount of collagen tissue (Figures 4(a) and 4(d)). After TNBS-ethanol enema, there was a small amount of collagen tissue on the surface of ulcerative and necrotic intestinal mucosal basal. More inflammatory cells infiltrated around the bronchia and collagen tissues were seen compared to the normal group, accompanied by red blood cell exudation and bronchial smooth muscle proliferation (Figures 4(b) and 4(e)). Four weeks later, profound collagen tissue proliferation were evident that even replaced the normal mucosa and glands in both the colon and lung tissue (Figures 4(c) and 4(f)). 

**Figure 5:**
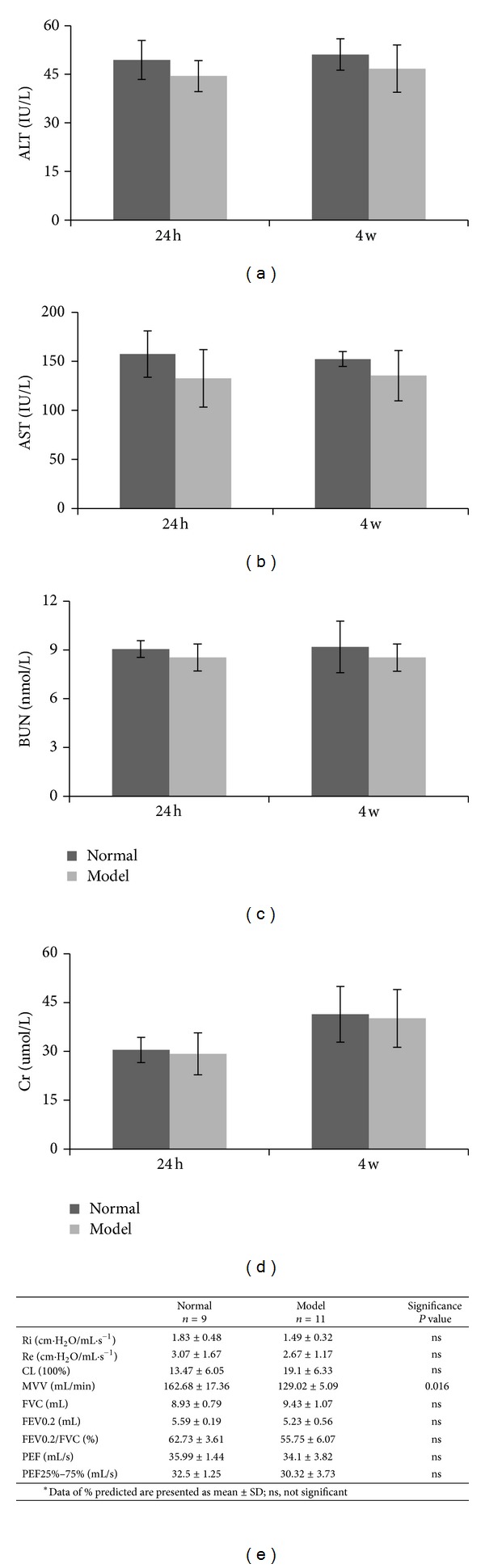
Liver, renal, and pulmonary function results. (a) and (b) Changes in ALT and AST of rat liver after TNBS-ethanol enema. (c) and (d) Changes in BUN, and Cr of rat liver after TNBS-ethanol enema. TNBS-ethanol enema resulted in a slightly decrease but no significant change in serum concentration of ALT, AST, BUN, and Cr when compared with the normal both in 24 hours and 4 weeks (*P* > 0.05). (e) Results of pulmonary function tests after TNBS-ethanol enema. Ri: resistance of airways at inspiration; Re: resistance of airways at expiration; CL: lung compliance; MVV: maximal voluntary ventilation; FVC: forced vital capacity; FEV0.2: forced expiratory volume in 0.2 second; FEV0.2/FVC: ration of FEV0.2 and FVC in %; PEF: peak expiratory flow; FEF25%–75%: and forced midexpiratory flow rate.

Functional abnormality was evidenced in the lung but not in the liver and kidney in immune-TNBS-ethanol-induced UC rat model.  Figure 5 illustrated the changes in the selected functional indices of rat liver, kidney, and lung after TNBS-ethanol enema. TNBS-ethanol enema resulted in a slightly but not statistically significant decrease in serum levels of ALT, AST, BUN, and Cr when compared with the normal (*P* > 0.05) at 24 hours and 4 weeks after TNBS-ethanol enema (Figures 5(a)–5(d)). On the other hand, pulmonary function test showed significantly decreased maximal voluntary ventilation (MVV) after TNBS-ethanol enema compared with the normal rats (*P* < 0.05), while no significant changes were seen in other parameters (Figure 5(e)).

### 3.3. Inflammation Triggers a Change in the Endothelium of the Intestinal and Pulmonary Vasculature Leading to Increased Adhesion Molecule Expression, Platelet Activation, Angiogenesis and Coagulation with Decreased Endothelial Barrier Functions. 

**Figure 6:**
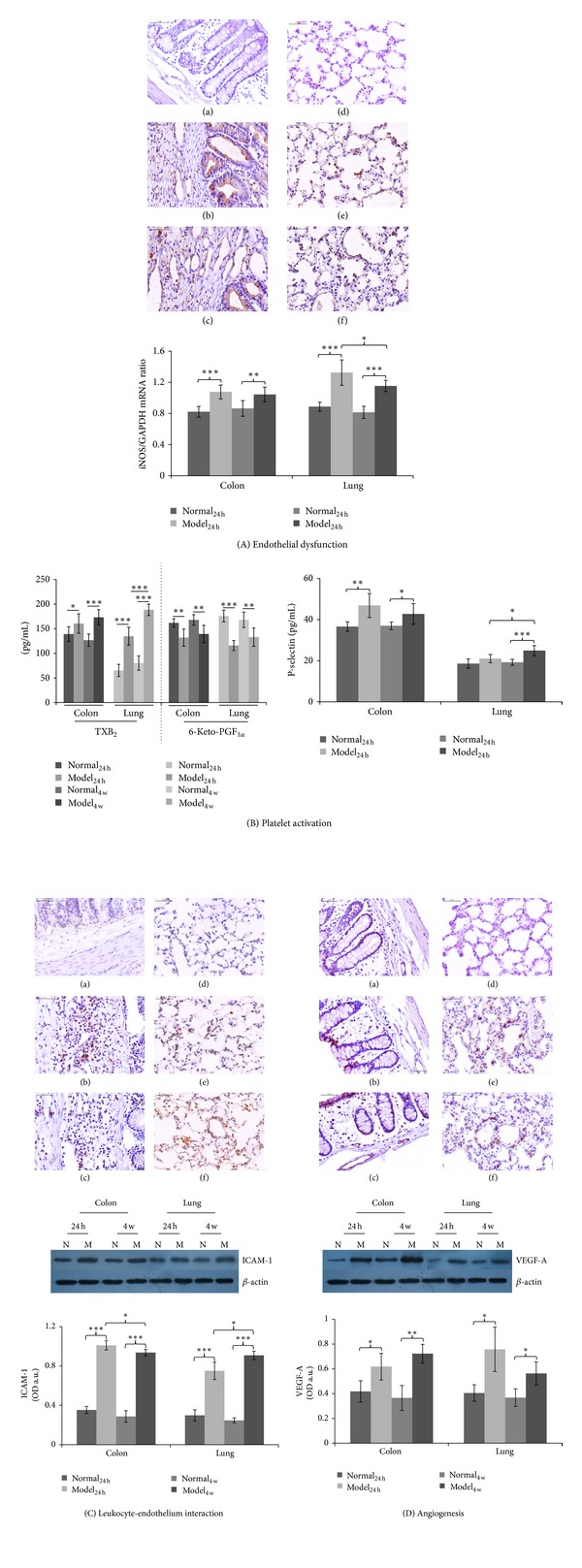
The role of microvasculature in the pathogenesis of ulcerative colitis. (A) Endothelial dysfunction: immunochemistry and RT-PCR results of iNOS in the colon and lung tissue homogenate. (B) Platelet activation: radioimmunoassay results of TXB_2_, 6-Keto-PGF_1*α*_ and P-selectin concentration (pg/mL) in the colon and lung tissue homogenate. (C) Leukocyte-endothelium interaction: Immunochemistry and western blotting results of ICAM-1 in the colon and lung tissue homogenate. (D) Angiogenesis: immunochemistry and western blotting results of VEGF-A in the colon and lung tissue homogenate. Band intensities were quantified using Image J software. (a) Normal colon, (b) model_24 h_ colon, (c) model_24 w_ colon, (d) normal lung, (e) model_24 h_ lung, and (f) model_24 w_ lung. Compared with the model group: *, *P* < 0.05; **, *P* < 0.01, ***; *P* < 0.001. Values are means, with error bars indicating SD.

Endothelial dysfunction: intrinsic alterations in the inflamed and remodeled microcirculation that underlie vascular dysfunction seem to play a fundamental role in deregulated inflammation, which characterizes UC. The expression of iNOS in the lung and colon tissue of animals was measured by immunochemistry and RT-PCR analysis (Figure 6(A)). There was no significant change of the iNOS concentration between normal and normal groups in the colon and lung tissue at both 24 hours and 4 weeks time points. Compared with the normal group, the colon tissue iNOS concentration of the model rats increased significantly (*P* < 0.01), especially 24 hours (*P* < 0.001). In the lung tissue, the iNOS concentration was also increased in the model group (*P* < 0.001). Changes in iNOS concentration seem more substantial at early time point than that at 4 weeks (*P* < 0.05).

Platelet activation: the intimate adherence of platelets to the endothelium is one of the characteristics of UC, and it is well established that platelets behave aberrantly in UC. Expression of TXB_2_, 6-Keto-PGF_1*α*_, and P-selectin in the lung and colon tissue of UC rats was measured by radioimmunoassay (Figure 6(B)). Results showed that the colon and lung tissue TXB_2_ and P-selectin expression of the model rats was significantly higher than the normal rats at both time points (*P* < 0.05), while the 6-Keto-PGF_1*α*_ expression of model was significantly lower than normal animals (*P* < 0.05).

Leukocyte-endothelium interaction: the inflamed endothelium displays enhanced leukocyte binding capacity and upregulation of adhesion molecules. ICAM-1 expression was measured with immunochemistry and western blotting (Figure 6(C)). ICAM-1 expression in both the colon and lung tissues of the model rats was higher than that in the normal rats (*P* < 0.001), within 24 hours. Concentration of ICAM-1 in the lung continuously increased within 4 weeks compared to that at 24 hours (*P* < 0.05).

Angiogenesis: the enhancement of angiogenesis in UC highlights neovascularization is a major contributor to the initiation and perpetuation of chronic intestinal inflammation. The expression of VEGF-A in the lung and colon tissue of UC rats was measured by immunochemistry and western blotting (Figure 6(D)). Results showed that the colon and lung tissue VEGF-A expression of the model rats was significantly higher than the normal rats in both 24 hours and 4 weeks (*P* < 0.05).

## 4. Discussion

A large number of epidemiological investigations [32, 33], case reports [34, 35], and clinical observation [36–38] have shown that the intestine diseases are often accompanied with pulmonary disorders and vice versa. The cellular and molecular mechanism underlying this phenomenon is that the complicated immunological, environmental, and genetic factors should trigger many cellular and molecular events that might be common in the two organs due to the same embryonic origin and similarity of tissue organization. Our present study is the first to demonstrate the close association between large intestine and lung in the immune-TNBS-ethanol-induced UC rat model. It combines immune stimulation, which contains some of the main factors of the clinical symptoms not a simple intestinal irritation. We have showed that both organs in the model animals shared common pathological changes including inflammatory cell infiltration, and increased collagen tissue. Our results also revealed for the first time that immune-TNBS-ethanol treatment in rats caused microvascular endothelium response towards inflammation characterized with endothelial dysfunction, platelet activation, leukocyte-endothelium interaction and angiogenesis in both the intestine and lung. Finally, we demonstrated that functions of both the intestine and lung were impaired in the above UC rats. Most importantly, we found that the association between the intestine and lung seems specific to the two organs in the disease model as we could not find any significant pathological or functional changes in other organs in the model animals. 

It is now clear that the abnormalities underlying UC pathogenesis are not only restricted to those mediated by classic immune cells, such as lymphocytes, macrophages, and dendritic cells but also involve nonimmune cells [39]. In particular, advances in vascular biology have outlined a central and multifaceted pathogenic role for the microcirculation in the initiation and perpetuation of UC [4, 40, 41]. Endothelial cells (ECs) play a crucial role in mucosal immune homeostasis by regulating the type and number of leukocytes migrating from the intravascular to the interstitial space, thus highlighting the endothelium as one of the pillars in inflammation pathogenesis [3, 5, 40, 41]. Indeed, the vascular response is a key component of inflammation, whereby ECs undergo activation in response to cytokines and bacterial products, leading to cell adhesion molecule expression, enhanced leukocyte interaction, and the influx of the inflammatory infiltrate into tissues and eventually angiogenesis [3, 5]. This is the first time that we demonstrated microvascular endothelium changes in response to the inflammation in both intestine and lung that led to increased angiogenesis, adhesion molecule expression, leukocyte extravasation, decreased endothelial barrier function, and increased coagulation. Endothelial activation and leukocyte interaction are thought to be critical regulatory steps in the initiation and maintenance of the inflammatory response. Many studies have shown that iNOS is predominantly expressed at sites of inflammation. Upregulation of iNOS has been found in experimental colitis [42–44]. Our finding of a strong positive correlation between inflamed score and intestinal/pulmonary iNOS expression in UC rats is in agreement with the above reports. Expression of iNOS in the colon and lung tissues may increase leukocyte adhesion [45]. Several cell adhesion molecules are involved in mediating leukocyte-endothelial interactions. It has been shown that selectins, integrins, and ICAM-1 are upregulated in inflamed endothelial cells to mediate a firm adhesion of leukocytes to activated endothelial cells [46, 47]. Our findings are in agreement with recent studies in ICAM-1 expressions of the colon, and lung tissues in the immune-TNBS-ethanol-induced UC rats are higher than that in the normal rats. In this model, the pathological changes in the colon and lung were synchronized after TNBS enema.

Patients suffering from exacerbations of UC have an increase in circulating platelet number [48]. Both leukocytes and platelets accumulate in the colonic microvasculature during experimental colitis, leading to microvascular dysfunction and tissue injury. A role for platelets in mediating leukocyte recruitment to the inflamed colon or lung is likely since platelet P-selectin, TXB_2_, and 6-Keto-PGF_1*α*_ are also localized to the intestinal or pulmonary microcirculation. 6-Keto-PGF_1*α*_ is thought to possess anti-inflammatory, immunosuppressive, and immunomodulatory actions. TXB_2_ enhances leucocyte recruitment and causes focal mucosal ischemia and injury through platelet activation and aggregation [49]. 

**Figure 7:**
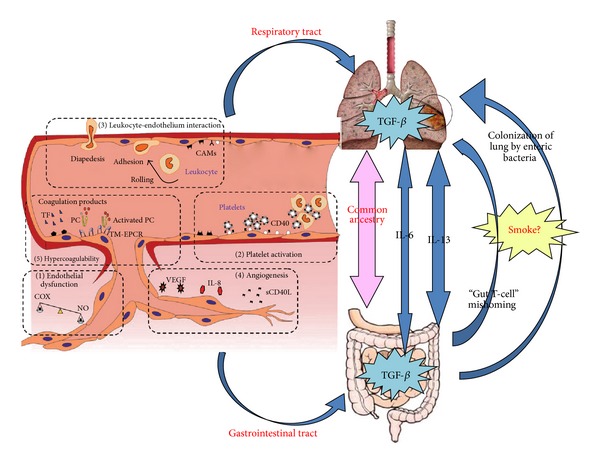
Possible microvasculature mechanism on how colitis affects lungs in the immune-TNBS-ethanol-induced UC rat model, perhaps related to the common ancestry of the bowel and the bronchial tree. Some etiological factors like smoke exposure may lead to gut-originating T-cell mishoming and promoting growth of enteric bacteria in the lung during inflammatory responses. Systemic IL-6, in conjunction with localized TGF-*β*, may drive cross-organ Th17-polarized inflammation. Systemic IL-13 may drive aberrant NKT and macrophage responses across organs. Inflammation triggers a change in the endothelium of the intestinal and pulmonary vasculature leading to increased adhesion molecule expression, platelet activation, angiogenesis, and coagulation with decreased endothelial barrier functions.

Our findings confirmed that immune-TNBS-ethanol can induce platelet activation, thromboxane synthesis, and blood hypercoagulability in the colon and lung tissues, resulting in the microcirculation thrombosis and microcirculatory dysfunction. One of the most novel aspects that directly implicate endothelial participation in inflammation is the process of angiogenesis [41]. It is now well established that angiogenesis and microvascular remodeling are intrinsic components of the tissue remodeling in chronic inflammatory diseases. Increasing evidence suggests that VEGF-A is one of the major proangiogenic factors involved in pathological angiogenesis. Our results identify VEGF-A as a molecule intimately involved in UC pathogenesis and one that acts at the crossroads between inflammatory-driven colonic and pulmonary angiogenesis and mucosal inflammation. This is consistent with Scaldaferri's study [5]. Based on these observations, our study has confirmed the communication between the large intestine and lung in UC rats and provided the experimental foundation for the theory of lung and large intestine that have a close internal connection (Figure 7).

In conclusion, this study provides evidence in support of immune-TNBS-ethanol-induced UC rat model as a useful tool to study possible association between the intestine and lung in ulcerative colitis. We identified a specific correlation in abnormalities in the large intestine and lung but not other organs in this model. Microvascular endothelium response towards inflammation by endothelial dysfunction, platelet activation, leukocyte-endothelium interaction, and angiogenesis may reflect a close relation in the large intestine and lung of this model. Our results suggest that different organs and tissues with the same embryonic origin may share the same pathological specificities in a disease. The present study provided a new way of thinking for pathological changes in clinical complex diseases manifested with multiorgan damage.

## Figures and Tables

**Table 1:** The sequences of the RT-PCR primers and cycling parameters.

Gene name	Sequence of primers (5′-3′)	Product size (bp)	Cycling parameters
iNOS	F: CCTCCTCCACCCTACCAAGT	199	10 min at 95°C; 40 cycles of 30 s at 95°C; 30 s at 60°C; 20 s at 72°C
	R: CACCCAAAGTGCTTCAGTCA		

GAPDH	F: CCATGGAGAAGGCTGGG	195	10 min at 95°C; 40 cycles of 30 s at 95°C; 30 s at 54°C; 20 s at 72°C
	R: CAAAGTTGTCATGGATGACC		

## References

[1] Baumgart DC, Carding SR (2007). Inflammatory bowel disease: cause and immunobiology. *Lancet*.

[2] Allez M, Modigliani R (2000). Clinical features of inflammatory bowel disease. *Current Opinion in Gastroenterology*.

[3] Chidlow JH, Greer JJM, Anthoni C (2009). Endothelial caveolin-1 regulates pathologic angiogenesis in a mouse model of colitis. *Gastroenterology*.

[4] Deban L, Correale C, Vetrano S, Malesci A, Danese S (2008). Multiple pathogenic roles of microvasculature in inflammatory bowel disease: a jack of all trades. *American Journal of Pathology*.

[5] Scaldaferri F, Vetrano S, Sans M (2009). VEGF-A links angiogenesis and inflammation in inflammatory bowel disease pathogenesis. *Gastroenterology*.

[6] Turner-Warwick M (1968). Fibrosing alveolitis and chronic liver disease. *The Quarterly Journal of Medicine*.

[7] Kraft SC, Earle RH, Roesler M, Esterly JR (1976). Unexplained bronchopulmonary disease with inflammatory bowel disease. *Archives of Internal Medicine*.

[8] Butland RJA, Cole P, Citron KM, Turner-Warwick M (1981). Chronic bronchial suppuration and inflammatory bowel disease. *Quarterly Journal of Medicine*.

[9] Camus P, Piard F, Ashcroft T, Gal AA, Colby TV (1993). The lung in inflammatory bowel disease. *Medicine*.

[10] Garg K, Lynch DA, Newell JD (1993). Inflammatory airways disease in ulcerative colitis: CT and high-resolution CT features. *Journal of Thoracic Imaging*.

[11] Higenbottam T, Cochrane GM, Clark TJ, Turner D, Millis R, Seymour W (1980). Bronchial disease in ulcerative colitis. *Thorax*.

[12] Sostegni R, Daperno M, Pera A (2007). Pulmonary manifestations of inflammatory bowel disease. *Digestive and Liver Disease*.

[13] Kuzela L, Vavrecka A, Prikazska M (1999). Pulmonary complications in patients with inflammatory bowel disease. *Hepato-Gastroenterology*.

[14] Marvisi M, Borrello PD, Brianti M, Fornarsari G, Marani G, Guariglia A (2000). Changes in the carbon monoxide diffusing capacity of the lung in ulcerative colitis. *European Respiratory Journal*.

[15] Songur N, Songur Y, Tuzun M (2003). Pulmonary function tests and high-resolution CT in the detection of pulmonary involvement in inflammatory bowel disease. *Journal of Clinical Gastroenterology*.

[16] Tzanakis N, Samiou M, Bouros D, Mouzas J, Kouroumalis E, Siafakas NM (1998). Small airways function in patients with inflammatory bowel disease. *American Journal of Respiratory and Critical Care Medicine*.

[17] Keely S, Talley NJ, Hansbro PM (2012). Pulmonary-intestinal cross-talk in mucosal inflammatory disease. *Mucosal Immunology*.

[18] Elson CO, Cong Y, Lorenz R, Weaver CT (2004). New developments in experimental models of inflammatory bowel disease. *Current Opinion in Gastroenterology*.

[19] Iqbal N, Oliver JR, Wagner FH, Lazenby AS, Elson CO, Weaver CT (2002). T helper 1 and T helper 2 cells are pathogenic in an antigen-specific model of colitis. *Journal of Experimental Medicine*.

[20] Ostanin DV, Bao J, Koboziev I (2009). T cell transfer model of chronic colitis: concepts, considerations, and tricks of the trade. *American Journal of Physiology*.

[21] Tahan G, Aytac E, Aytekin H (2011). Vitamin E has a dual effect of anti-inflammatory and antioxidant activities in acetic acid-induced ulcerative colitis in rats. *Canadian Journal of Surgery*.

[22] Te Velde AA, De Kort F, Sterrenburg E (2007). Comparative analysis of colonic gene expression of three experimental colitis models mimicking inflammatory bowel disease. *Inflammatory Bowel Diseases*.

[23] Yukitake H, Kimura H, Suzuki H (2011). BTZO-15, an ARE-activator, ameliorates DSS-and TNBS-induced colitis in rats. *PLoS ONE*.

[24] Auli M, Nasser Y, Ho W (2008). Neuromuscular changes in a rat model of colitis. *Autonomic Neuroscience*.

[25] Kazi H, Qian Z (2009). Crocetin reduces TNBS-induced experimental colitis in mice by downregulation of NFkB. *Saudi Journal of Gastroenterology*.

[26] Te Velde AA, Verstege MI, Hommes DW (2006). Critical appraisal of the current practice in murine TNBS-induced colitis. *Inflammatory Bowel Diseases*.

[27] Dohi T, Fujihashi K (2006). Type 1 and 2 T helper cell-mediated colitis. *Current Opinion in Gastroenterology*.

[28] Liu Y, Hua Q, Lei H, Li P (2011). Effect of Tong Luo Jiu Nao on Abeta-degrading enzymes in AD rat brains. *Journal of Ethnopharmacology*.

[29] Zhu MY, Lu YM, Ou YX, Zhang HZ, Chen WX (2012). Dynamic progress of 2,4,6-trinitrobenzene sulfonic acid induced chronic colitis and fibrosis in rat model. *Journal of Digestive Diseases*.

[30] Liu Y, Yang G, Bu X (2011). Cell-type-specific regulation of raft-associated Akt signaling. *Cell Death and Disease*.

[31] Schnupf P, Sansonetti PJ (2012). Quantitative RT-PCR profiling of the rabbit immune response: assessment of acute Shigella flexneri infection. *PLoS ONE*.

[32] Larsen S, Bendtzen K, Nielsen OH (2010). Extraintestinal manifestations of inflammatory bowel disease: epidemiology, diagnosis, and management. *Annals of Medicine*.

[33] Raj AA, Birring SS, Green R, Grant A, de Caestecker J, Pavord ID (2008). Prevalence of inflammatory bowel disease in patients with airways disease. *Respiratory Medicine*.

[34] Chikano S, Sawada K, Ohnishi K, Fukunaga K, Tanaka J, Shimoyama T (2001). Interstitial pneumonia accompanying ulcerative colitis. *Internal Medicine*.

[35] Marten K, Fend F, Hautmann H, Kremer M, Rummeny EJ, Engelke C (2005). Fatal acute exacerbation of usual interstitial pneumonia in ulcerative colitis. *British Journal of Radiology*.

[36] Kelly MG, Frizelle FA, Thornley PT, Beckert L, Epton M, Lynch AC (2006). Inflammatory bowel disease and the lung: is there a link between surgery and bronchiectasis?. *International Journal of Colorectal Disease*.

[37] Sun HY, Wang XY, Wu J (2011). The interior-exterior correlation between fei and dachang from the lung function injury in ulcerative colitis patients. *Zhongguo Zhong Xi Yi Jie He Za Zhi*.

[38] Yilmaz A, Demirci NY, Hoşgün D (2010). Pulmonary involvement in inflammatory bowel disease. *World Journal of Gastroenterology*.

[39] Fiocchi C (1997). Intestinal inflammation: a complex interplay of immune and nonimmune cell interactions. *American Journal of Physiology*.

[40] Binion DG, Rafiee P (2009). Is Inflammatory bowel disease a vascular disease? Targeting angiogenesis improves chronic inflammation in inflammatory bowel disease. *Gastroenterology*.

[41] Cromer WE, Mathis JM, Granger DN, Chaitanya GV, Alexander JS (2011). Role of the endothelium in inflammatory bowel diseases. *World Journal of Gastroenterology*.

[42] da Silva MS, Sánchez-Fidalgo S, Talero E (2010). Anti-inflammatory intestinal activity of *Abarema cochliacarpos* (Gomes) Barneby & Grimes in TNBS colitis model. *Journal of Ethnopharmacology*.

[43] Krieglstein CF, Anthoni C, Cerwinka WH (2007). Role of blood- and tissue-associated inducible nitric-oxide synthase in colonic inflammation. *American Journal of Pathology*.

[44] Talero E, Sánchez-Fidalgo S, de la Lastra CA, Illanes M, Calvo JR, Motilva V (2008). Acute and chronic responses associated with adrenomedullin administration in experimental colitis. *Peptides*.

[45] Hickey MJ (2001). Role of inducible nitric oxide synthase in the regulation of leucocyte recruitment. *Clinical Science*.

[46] Abdallah DM, Ismael NR (2011). Resveratrol abrogates adhesion molecules and protects against TNBS-induced ulcerative colitis in rats. *Canadian Journal of Physiology and Pharmacology*.

[47] Thomas S, Baumgart DC (2012). Targeting leukocyte migration and adhesion in Crohn's disease and ulcerative colitis. *Inflammopharmacology*.

[48] Vowinkel T, Wood KC, Stokes KY (2007). Mechanisms of platelet and leukocyte recruitment in experimental colitis. *American Journal of Physiology*.

[49] Auwerda JJA, Zijlstra FJ, Tak CJAM, Van den Ingh HFGM, Wilson JHP, Ouwendijk RJT (2001). Ridogrel enemas in distal ulcerative colitis. *European Journal of Gastroenterology and Hepatology*.

